# Brain microstructure alterations in subjective cognitive decline: a multi-component T2 relaxometry study

**DOI:** 10.1093/braincomms/fcaf017

**Published:** 2025-01-16

**Authors:** Miguel Ángel Rivas-Fernández, Mustapha Bouhrara, Erick J Canales-Rodríguez, Mónica Lindín, Montserrat Zurrón, Fernando Díaz, Santiago Galdo-Álvarez

**Affiliations:** Division of Endocrinology, Diabetes and Metabolism, Children’s Hospital of Los Angeles, Los Angeles, CA 90027, USA; Laboratory of Clinical Investigation, National Institute on Aging, National Institutes of Health, Baltimore, MD 21224, USA; Department of Radiology, Centre Hospitalier Universitaire Vaudois (CHUV), Lausanne, CH-1011, Switzerland; Computational Medical Imaging and Machine Learning Section, Center for Biomedical Imaging (CIBM), Lausanne, CH-1015, Switzerland; Signal Processing Laboratory (LTS5), École Polytechnique Féderale de Lausanne (EPFL), Laussane, CH-1015, Switzerland; Department of Clinical Psychology and Psychobiology, Universidade de Santiago de Compostela (USC), Santiago de Compostela 15782, Spain; Applied Cognitive Neuroscience and Psychogerontology Research Group (Neucoga-Aging), Instituto de Psicoloxía, USC (IPsiUS), Santiago de Compostela, 15782, Spain; Cognitive Neuroscience Research Group, Health Research Institute of Santiago de Compostela (IDIS), Santiago de Compostela, 15706, Spain; Department of Clinical Psychology and Psychobiology, Universidade de Santiago de Compostela (USC), Santiago de Compostela 15782, Spain; Applied Cognitive Neuroscience and Psychogerontology Research Group (Neucoga-Aging), Instituto de Psicoloxía, USC (IPsiUS), Santiago de Compostela, 15782, Spain; Cognitive Neuroscience Research Group, Health Research Institute of Santiago de Compostela (IDIS), Santiago de Compostela, 15706, Spain; Department of Clinical Psychology and Psychobiology, Universidade de Santiago de Compostela (USC), Santiago de Compostela 15782, Spain; Applied Cognitive Neuroscience and Psychogerontology Research Group (Neucoga-Aging), Instituto de Psicoloxía, USC (IPsiUS), Santiago de Compostela, 15782, Spain; Cognitive Neuroscience Research Group, Health Research Institute of Santiago de Compostela (IDIS), Santiago de Compostela, 15706, Spain; Department of Clinical Psychology and Psychobiology, Universidade de Santiago de Compostela (USC), Santiago de Compostela 15782, Spain; Applied Cognitive Neuroscience and Psychogerontology Research Group (Neucoga-Aging), Instituto de Psicoloxía, USC (IPsiUS), Santiago de Compostela, 15782, Spain; Cognitive Neuroscience Research Group, Health Research Institute of Santiago de Compostela (IDIS), Santiago de Compostela, 15706, Spain

**Keywords:** myelin content, intra-extracellular water fraction, neuroinflammation, neuromicrostructural tissue integrity

## Abstract

Previous research has revealed patterns of brain atrophy in subjective cognitive decline, a potential preclinical stage of Alzheimer’s disease. However, the involvement of myelin content and microstructural alterations in subjective cognitive decline has not previously been investigated. This study included three groups of participants recruited from the Compostela Aging Study project: 53 cognitively unimpaired adults, 16 individuals with subjective cognitive decline and hippocampal atrophy and 70 with subjective cognitive decline and no hippocampal atrophy. Group differences were analysed across five MRI biomarkers derived from multi-component T2 relaxometry, each sensitive to variations in cerebral composition and microstructural tissue integrity. Although no significant differences in myelin content were observed between groups, the subjective cognitive decline with hippocampal atrophy group exhibited a larger free-water fraction, and reduced fraction and relaxation times of the intra/extracellular water compartment in frontal, parietal and medial temporal lobe brain regions and white matter tracts as compared with the other groups. Moreover, both subjective cognitive decline groups displayed lower total water content as compared with the control group and the subjective cognitive decline with hippocampal atrophy group showed lower total water content as compared with the subjective cognitive decline without hippocampal atrophy group. These changes are likely related to microstructural tissue differences related to neuroinflammation, axonal degeneration, iron accumulation or other physiologic variations, calling for further examinations.

## Teaser

Cerebral composition and microstructural tissue integrity in individuals with subjective cognitive decline were examined. Although no group differences in myelin content were found, participants with evidence of hippocampus atrophy exhibited altered water compartment biomarkers, suggesting neuroinflammation, axonal degeneration, or iron deposition, highlighting microstructural changes potentially linked to Alzheimer’s disease progression.

## Graphical Abstract

**Graphical abstract:**
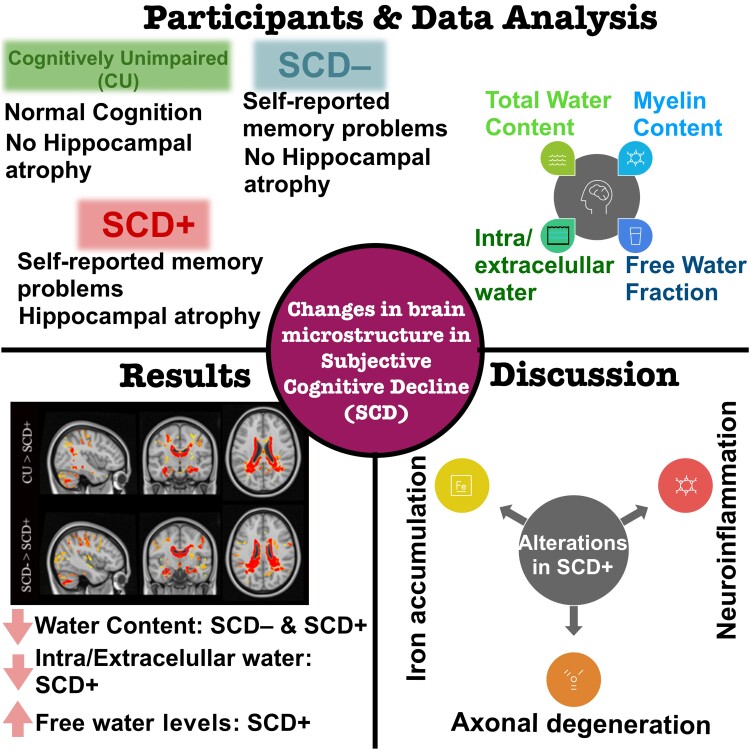

## Introduction

The ageing population has led to a surge in age-related diseases, including Alzheimer’s disease. Research has shown that pathophysiological changes associated with Alzheimer’s disease-related dementia can occur years before the onset of clinical symptoms.^1^ As a result, subjective cognitive decline (SCD) has been identified as a preclinical stage of Alzheimer’s disease, characterized by normal cognition but subjective cognitive complaints (SCCs).^2,3^ Individuals with SCD face twice the risk of developing dementia than those without such complaints.^4^ Moreover, individuals who progress to Alzheimer’s disease dementia may first experience mild cognitive impairment (MCI), a prodromal stage marked by objective evidence of cognitive decline but with preserved daily functioning.^5,6^

The SCD initiative (SCD-I) working group identified a set of features, known as SCD plus (SCD+) criteria,^3^ which may increase the risk of developing Alzheimer’s disease-related dementia in individuals with SCD. Some of these features include the genetic risk factor APOℇ4 allele, or evidence of brain atrophy in medial temporal lobe areas, particularly the hippocampus.^3^ Additionally, neuroimaging studies have focused on establishing non-invasive markers of brain structural integrity for preclinical conditions such as SCD. Evidence from brain morphometry and diffusion tensor imaging (DTI) studies shows that individuals with SCD exhibit grey matter atrophy in medial temporal, frontal and parietal lobes,^7,8^ as well as white matter alterations, in the corpus callosum, corona radiata and longitudinal fasciculus.^9-11^ However, MRI techniques such as DTI are of limited use in quantifying specific determinants of cerebral tissue, which can be influenced by several factors, including axonal packing density, axonal membrane status and myelin thickness.^12^ Multi-component T2-relaxometry has emerged as a promising complementary MRI technique for evaluating tissue water compartments and extracting quantitative data on multi-compartmental transverse relaxation times and corresponding water fractions,^13^ which can be used as proxies for the biological properties of brain tissues.^14^

Multi-component T2-relaxometry allows estimation of the intra-extracellular water fraction (IEWF) and the geometric mean of the intracellular and extracellular water (T2^IE^), which, respectively, represent the amount of water and associated transverse relaxation in the intracellular and extracellular space. The IEWF and T2^IE^ parameters are negatively associated with age^15,16^ and reduced values in these metrics have been proposed as suggestive biomarkers of neuronal degeneration^15^ and increased iron deposition,^17^ respectively. Another important derived parameter is the free/quasi-free water fraction (FQFWF), representing the amount of the most freely relaxing water pool within a given voxel. Higher FQFWF values are indicators of potential neuroinflammation and neurodegeneration.^18,19^ The analysis also estimates the total water content (TWC), calculated as the total area under the T2 distribution curve, which serves as a proxy for the water content within a given voxel.^20^ TWC maps are useful to support IEWF changes^16^ as reductions in the IEWF compartment can be partially attributed to an increase in the FQFWF biomarker or in the myelin water fraction (MWF), a biomarker reflecting the area under the curve of the fast-relaxing water compartment normalized by the TWC. The MWF is a marker that is strongly correlated with myelin content, as demonstrated in previous studies.^21-23^ In the context of Alzheimer’s disease research, evidence has shown that individuals with MCI or Alzheimer’s disease dementia display increased free water in the corticospinal tract, the cingulum bundle and the fornix^19^ as well as reduced MWF in the corpus callosum and the frontal and posterior portions of the lateral ventricles.^24-26^

To our knowledge, myelin content and microstructural alterations remain unexplored in SCD. Investigating differences in these advanced imaging biomarkers is paramount not only for evaluating their sensitivity in capturing early structural changes preceding clinical symptoms, but for informing strategies for early interventions. The main objective of the present study is to assess cognitive and microstructural differences between cognitively unimpaired (CU) with SCD individuals, either with or without increased Alzheimer’s disease dementia risk (SCD+, SCD−). The group comparisons focused on cognitive performance as well as imaging markers of microstructural integrity of cerebral tissue as derived from the multi-component relaxometry: MWF, IEWF, T2^IE^, TWC and FQFWF. Group differences were assessed both at the whole-brain level as well as in brain regions known for their higher vulnerability to Alzheimer’s disease pathophysiology (i.e. Alzheimer’s disease signature^27^). While we did not expect group differences in cognitive performance, we anticipated that individuals with SCD would display differences in some of these biomarkers as compared with the control group.

## Materials and methods

### Participants

A total of 139 volunteers were recruited as a part of the Compostela Aging Study (CompAS), an ongoing longitudinal project^28^ aimed at the early detection and progression of cognitive impairment in patients aged 50+ years with SCCs attending Primary Care Health Centres in Galicia, Spain. Participants were referred to the project by general practitioners and excluded if they had any of the following: (i) previous diagnosis of neurological or psychiatric disease, specifically dementia; (ii) clinical stroke or severe cardiovascular disease; (iii) previous chemotherapy or cancer treatment; (iv) non-compensable motor-sensory impairment; (v) history of brain damage or brain surgery; (vi) uncontrolled Type II diabetes mellitus: and (vii) substance (alcohol or drug) abuse/dependency. The study was approved by the Galician Clinical Research Ethics Committee (Xunta de Galicia, Spain; Refs. 2017/498; 2022/116) and was performed in accordance with the ethical standards established in the 1964 Declaration of Helsinki.^29^ All participants gave their written informed consent prior to taking part in the study.

### Neuropsychological assessment

Participants underwent comprehensive clinical and neuropsychological assessment. In order to evaluate general cognitive functioning, all participants completed the Spanish version of the Mini Mental State Examination (MMSE).^30^ In addition, the Spanish version of the Geriatric Depression Scale (GDS-15)^31^ was administered to evaluate depressive symptoms.^32^

The following cognitive domains were also evaluated: (i) attention, with the trail making test A,^33^ which assesses attentional visual-perceptive searching and perceptive-motor processing speed, and with the Attention and Calculation CAMCOG-R subscale (Cambridge Cognitive Assessment-Revised), which assesses attentional control^34^; (ii) executive functioning, with the trail making test B,^33^ which evaluates working memory and cognitive flexibility,^35^ the phonological verbal fluency test (say words starting with ‘p’ in 1 min), which assesses working memory and inhibition,^36^ and the executive function CAMCOG-R subscale, which assesses abstract thinking and categorization; (iii) memory, with the List A total recall (immediate memory of words), the long-delay free recall (long term verbal memory of words) from the California verbal learning test (CVLT)^37^ and the Memory CAMCOG-R subscale, which together provide a joint measure composed by short delay visual memory for objects and recognition, and recent and remote memory. To evaluate language processes, we used the Boston naming test (BNT),^38^ the Spanish version of the semantic verbal fluency (animals)^36^ and the Language CAMCOG-R subscale, which together provide a joint measure of oral comprehension, repetition, naming and reading comprehension. Finally, the Lawton and Brody index (maximum possible score = 8) was used to evaluate instrumental activities of daily living.^39^ This comprehensive assessment was conducted by trained psychologists with expertise in ageing and dementia and participants were classified at a special meeting of the research team as CU or SCD.

In addition to this comprehensive neuropsychological assessment, for each participant, we estimated the degree of normative deviation (*Z*-scores) of the hippocampal volume from the reference population as this structural metric has been proposed as a potential biomarker of Alzheimer’s disease.^3^ To estimate the adjusted *Z*-scores of hippocampal volume, cortical reconstruction was conducted with the T1-weighted 3D MPRAGE image of the brain of each participant by means of the automated pre-processing pipeline (*recon-all*) implemented in FreeSurfer 6.0 software (https://surfer.nmr.mgh.harvard.edu/). A quality control protocol was conducted over the FreeSurfer segmentations with the Freeview program. Segmentations were visually inspected on a slice-by-slice basis by an experienced technician to enhance the reliability of pial and white matter surfaces. All manual editions were conducted following the technical instructions included in the Freeview Guide (https://surfer.nmr.mgh.harvard.edu/fswiki/FreeviewGuide). For each measure in each participant, the degree of deviation was then compared with a normative model consisting of 6909 CU individuals aged 18 to 100 years.^40^ This normative model has been used in previous studies,^41,42^ and the cut-off criteria for determining whether hippocampal volume estimates are within the normal range are consistent with those used in earlier Alzheimer’s disease research.^43^ Participants were diagnosed as having an increased Alzheimer’s disease risk if they had adjusted *Z*-scores in the left and/or right hippocampal volume below one standard deviation (*Z* < −1) according to their age, sex and intracranial volume.

Participants with normal general cognitive performance, tested by the Spanish version^44^ of the Cambridge Cognitive Assessment—Revised battery (CAMCOG-R)^34^ according to age and education norms^45^ and who did not meet the aforementioned Alzheimer’s disease risk criteria were classified as CU. All CU participants had adjusted *Z*-scores of hippocampal volume within the normal range according to their age, sex and intracranial volume. Diagnosis of SCD followed the two main criteria proposed by the SCD-I Working Group^3^: (i) self-experienced persistent decline in cognitive capacity, especially in memory, relative to a previously normal cognitive status, which is unrelated to an acute event; and (ii) normal cognitive performance in standardized tests adjusted for age and education. For this purpose, participants were asked about their possible self-experienced persistent decline in cognitive capacity and to express their worries about failures in attention and memory in the last few years. Possible known explanations for these complaints were ruled out through the initial interview. Normal cognitive status was verified by the same procedure for the CU participants. In addition, considering the presence of cognitive complaints in normative ageing,^46^ we took into account the severity of concern^2^ and participants were only identified as SCD when the complaints exceeded the score in the Questionnaire d’auto-évaluation de la Mémoire (QAM)^47,48^ that corresponds to the fifth percentile according to age norms.^49^ The cut-off point of the QAM short version, set at the fifth percentile of total scoring adjusted for age, has proven valid in assessing the severity of SCCs and predicting progression from SCD to MCI and dementia, as well as from MCI to dementia.^49^ In addition to the two main diagnostic criteria proposed by the SCD-I Working Group,^3^ we assessed whether the volume of the left and/or right hippocampus in all participants with SCD was within the normal range according to their age, sex and intracranial volume or was more than one standard deviation below this level. In line with previous studies that used the same criteria for enhanced hippocampal atrophy,^43^ participants with SCD who had a left and/or right hippocampal volume one standard deviation below that of a normative sample were diagnosed as SCD plus (SCD+) and participants who had normal hippocampal volumes were diagnosed as SCD minus (SCD−).

The three groups evaluated were matched in gender and years of education but not in age. In addition, there was a significant group difference in depressive symptoms. Thus, age and GDS scores were included as covariate in all statistical analyses. Demographics and between-group differences in neuropsychological measures are summarized in Table 1.

**Table 1:** Demographic and clinical characteristics of participants groups

	CU *N* = 53	SCD− *N* = 70	SCD+ *N* = 16	*F*	*P =* *
**Age**	63.26 (6.58)	65.86 (7.82)	68.35 (7.47)	3.60	0.03
**Years of education**	14.30 (5.24)	13.44 (5.24)	14.63 (6.45)	0.54	0.58
**Gender (female/male)**	40/13	54/16	10/6		0.47 a
**GDS score**	2.17 (3.02)	3.27 (2.86)	3.88 (2.92)	3.09	0.05
**SCCs**					
Patient	14.94 (1.92)	20.54 (2.40)	20.00 (1.97)	103.26	<0.001
Informant-caregiver	5.85 (5.65)	7.78 (6.08)	8.63 (7.62)	0.89	0.41
**General functioning**					
MMSE	28.93 (1.39)	28.81 (1.30)	28.25 (1.34)	0.29	0.75
**Attention**					
TMT-A (seconds)	38.02 (11.27)	45.59 (18.50)	47.06 (26.06)	1.20	0.30
CAMCOG-R (Attention and Calculation)	8.26 (1.08)	8.10 (1.23)	7.56 (1.83)	0.03	0.97
**Executive function**					
TMT-B (seconds) b	87.89 (43.56)	98.03 (50.04)	109.13 (54.32)	0.05	0.95
Phonological verbal fluency	16.77 (4.89)	15.86 (4.81)	15.75 (6.01)	0.13	0.88
CAMCOG-R (Executive Function)	22.94 (3.26)	21.86 (4.15)	21.31 (4.85)	0.27	0.76
**Memory**					
CVLT (long-delay free recall)	13.19 (2.65)	12.66 (3.03)	10.88 (3.26)	1.03	0.36
CVLT (List A total recall)	57.04 (9.06)	56.91 (10.83)	52.44 (9.45)	1.08	0.34
CAMCOG-R (Memory)	23.45 (2.00)	22.09 (2.79)	22.19 (2.83)	2.57	0.08
**Language**					
BNT	56.43 (4.16)	54.53 (5.23)	54.38 (7.33)	0.73	0.48
Semantic verbal fluency (animals)	23.06 (5.85)	20.66 (5.73)	20.50 (6.81)	0.81	0.44
CAMCOG-R (Language)	28.17 (1.55)	27.84 (1.70)	27.56 (2.13)	0.09	0.91

Mean values and standard deviations (SD in brackets) of demographic and neuropsychological measures in the CU (cognitively unimpaired individuals) and the SCD− and SCD+ groups.

MANOVA with age and GDS scores as covariates; *P < 0.05. GDS, geriatric depression scale; MMSE, Mini-Mental State Examination; TMT-A/B, trail making test (version A/B); CVLT, California verbal learning test; CAMCOG-R, Cambridge cognitive examination; BNT, Boston naming test; SCD, subjective cognitive decline.

^a^χ^2^ test.

^b^One participant did not complete the TMT-B test.

### MRI data acquisition

MRI was performed with a Philips 3T Achieva scanner (Philips Medical System, Best, The Netherlands). Three types of MRI sequences were acquired: a sagittal T1-weighted using the 3D MPRAGE sequence (repetition time/echo time = 7.45 ms/3.40 ms, flip angle = 8°; 180 slices, voxel size = 1 × 1 × 1 mm, field of view = 240 × 240 mm^2^, matrix size = 240 × 240 mm; slice-thickness = 1 mm), axial multi-spin-echo images using the 3D Gradient Spin Echo (GRASE) sequence (repetition time = 2000 ms, number of echoes = 32, ΔTE = 10.24 ms, flip angle 90°, voxel size = 1.7 × 1.7 × 4 mm, field-of-view = 180 × 219 × 166 mm, matrix size = 108 × 125 × 83) and a sagittal 3D Fluid Attenuated Inversion Recovery (FLAIR) sequence (repetition time/echo time = 4800 ms/300.85, flip angle = 90°, 321 slices, voxel size = 0.49 × 0.49 × 0.56, field of view = 250 × 250 mm^2^, matrix size = 512 × 512 mm, slice thickness = 1.12 mm). Participants were provided with foam earplugs to attenuate scanner noise, while head motion was minimized by using a head restraint system by placing foam padding around the subject’s head.

### MRI data analysis

#### Estimation of MRI relaxometry parameters

The 3D GRASE MRI brain data were denoised using the nonlocal estimation of multi-spectral magnitudes method^50^ and corrected for Gibbs ringing artefacts with the *mrdegibbs* utility implemented in the MRtrix 3.0.3v software.^51^ The preprocessed data were then analysed using the Multi-component T2 reconstruction toolbox,^52^ publicly available at https://github.com/ejcanalesr/multicomponent-T2-toolbox. The extended phase graph model^53^ was used to generate different dictionary matrices of synthetic signals for various flip angles, for a set of *N* = 60 logarithmically spaced T2 values ranging from 10 to 2000 ms.^54^ The standard non-negative least squares (NNLS) algorithm was used to fit the data to these dictionary matrices. For each voxel, the optimal flip angle was determined by minimizing the mean square error between the measured and predicted signals.^53^ For the sole purpose of determining the optimal flip angle and associated dictionary, the data were smoothed with a Gaussian kernel [full width at half maximum (FWHM) of 4.8 mm] prior to the NNLS fitting, following a previous recommendation.^55^

The intra-voxel T2 distribution was then calculated using the regularized NNLS method,^23,56^ with the dictionary matrix corresponding to the optimal flip angle and an identity matrix in the Tikhonov regularization term to promote smooth T2 distributions. The optimal regularization parameter was determined using the *X*^2^ residual fitting criterion,^57,58^ specifically the *X*^2^-I method described by Canales-Rodríguez *et al*.^52,59^ with parameter *k* = 1.02.^23,56^

We estimated five quantitative metrics from T2 distributions. First, the TWC was calculated as the area under the entire T2 distribution curve. To enhance comparability, TWC maps were normalized to cerebrospinal fluid (CSF), assuming similar CSF composition across groups. This normalization leveraged CSF’s stable characteristics as an internal reference, providing a reliable baseline for comparison. Second, the MWF was calculated as the area under the curve for T2 times smaller than the myelin water cut-off (T2 < 40 ms), normalized by TWC.^20^ MWF is a neuroimaging marker strongly correlated with myelin content.^21-23^ Third, the IEWF was computed as the area under the T2 distribution in the range 40–200 ms, normalized by TWC. Fourth, the T2 of the intracellular and extracellular water (T2^IE^) was computed as the geometric mean of the distribution in the T2 range of 40–200 ms.^60^ The IEWF and the T2^IE^ represent the amount of water and associated transverse relaxation in the intracellular and extracellular spaces. Finally, the FQFWF was estimated as the area under the T2 spectrum in the range 200–2000 ms, normalized by TWC.^15^ Free water is seen as a neuroimaging proxy of neuroinflammation and neurodegeneration.^18,19^ Table 2 provides technical and physiologic descriptions of these MRI biomarkers.

**Table 2:** Summary of the multi-component T2 relaxometry metrics evaluated, their descriptors and suspected aetiology

Biomarker	Descriptor	Suspected aetiology
TWC	Area under the curve of the whole T2 distribution.	Neuroimaging proxy for the TWC within a given voxel.
MWF	Area under the curve of the fast-relaxing water component normalized by the TWC.	Decreased MWF is a marker of demyelination.
IEWF	Area under the curve of the slowly relaxing water components normalized by the TWC.	Reduced IEWF is a marker of a reduction in the relative volume of the intra- and extra-axonal compartment, suggesting neuronal degeneration.
T2^IE^	Transverse relaxation time of the slowly relaxing water component corresponding to the intracellular and extracellular waters.	Reduced T2^IE^ has been suggested as a putative biomarker of iron accumulation.
FQFWF	Fraction of the free water relative to TWC.	Higher values are associated with neuroinflammation.

TWC, total water content; MWF, myelin water fraction; IEWF, intra-extracellular water fraction; T2IE, geometric mean of intra-extracellular water; FQFWF, free-quasi-free water fraction.

#### Image registration and region-of-interest determination

For each participant, the GRASE scans were registered to the T1-weighted MPRAGE scans.^61^ Then a spatial nonlinear normalization of the corresponding T1-weighted MPRAGE image to the Montreal Neurological Institute (MNI) standard space was performed using the symmetric normalization method, as implemented in the advanced normalization tools (ANTs).^61^ The derived deformation fields were then used to normalize the parameter maps to the MNI standard space. These maps were spatially smoothed with a Gaussian kernel of 2 mm FWHM. The Automated Anatomical Labelling and the Johns Hopkins University atlases were employed for anatomical reference of grey matter regions and white matter tracts, respectively.^62,63^

Mean values of each MRI parameter in specific region-of-interest (ROIs) were also calculated to evaluate group differences in the cortical Alzheimer’s disease signature.^27^ To this end, binary masks of the entorhinal cortex, inferior temporal gyrus, middle temporal gyrus, inferior parietal lobe, fusiform gyrus and precuneus were generated using the cortical segmentation obtained with FreeSurfer’s cortical reconstruction, and the MRI parameter values were estimated in these ROIs.

#### White matter hyperintensities

A global estimate of the WMH volume of each participant was obtained using the lesion prediction algorithm implemented in the Lesion Segmentation toolbox^64^ for Statistical Parametric Mapping (https://www.fil.ion.ucl.ac.uk/spm/software/spm12/), with the FLAIR images registered to the T1w images as input. The extracted WMH volumes were normalized by the total intracranial volume of each participant and were then log transformed to normalize the population variance. The probability lesion maps were binarized with a threshold of 0.5 to reduce the false positive rate (i.e. voxels with a probability above 0.5 were considered lesions, and voxels with a probability below 0.5 were set to 0). Once the binary lesion maps were constructed, FLAIR images were registered to the GRASE images using ANTs. The transformation matrix was then applied to the binary lesion maps.

### Statistical analysis

#### Neuropsychological data

Multi-variate analysis of variance (MANOVA) was used to evaluate the effect of group on cognitive performance, by including the scores of neuropsychological tests as dependent variables and age and the GDS scores as covariates. The Bonferroni method was used to correct for multiple comparisons, and the significance level was set at *P* < 0.05. All statistical analyses were performed with SPSS Statistics for Windows, version 26 (SPSS Inc., Chicago, Ill, USA).

#### MRI relaxometry data

A voxel-wise analysis was conducted to assess differences in the parameters evaluated. Group differences between the three groups were evaluated using ANCOVA adjusting for age, GDS scores and the global WMHs volume estimates as covariates. Significant association was then followed by an *ad hoc* analysis consisting of pairwise comparisons using two sample *t*-tests. All statistical analyses were conducted using the randomize FSL tool^65^ by applying the threshold-free cluster enhancement method^66^ with 10 000 permutations. To evaluate group-differences in the cortical Alzheimer’s disease signature, a multi-variate general linear model was constructed, with Group as the fixed factor, the Alzheimer’s disease signature of each metric as dependent variables and age and the GDS scores as covariates. In order to evaluate whether microstructural differences in the hippocampus revealed by the whole brain analysis are driven by the hippocampal atrophy differences, a univariate general linear model was constructed for each hippocampus with Group as the fixed factor, the estimates of FQFWF, IEWF and T2^IE^ of the left/right hippocampus as dependent variables and also age, GDS scores and the *Z*-scores of the left/right hippocampus as covariates. Bonferroni correction was used for multiple comparisons, and the significance level was set at *P* < 0.05.

## Results

### Neuropsychological data

Although there were no significant differences between groups in neuropsychological tests, a group difference in depressive symptomatology (*F* = 3.09; *P* = 0.049) was observed, but pairwise comparisons did not survive multiple comparisons correction. As expected, the frequency of SCCs was higher in both SCD groups as compared with CU.

### MRI relaxometry data

#### Whole brain analysis

**Figure 1:**
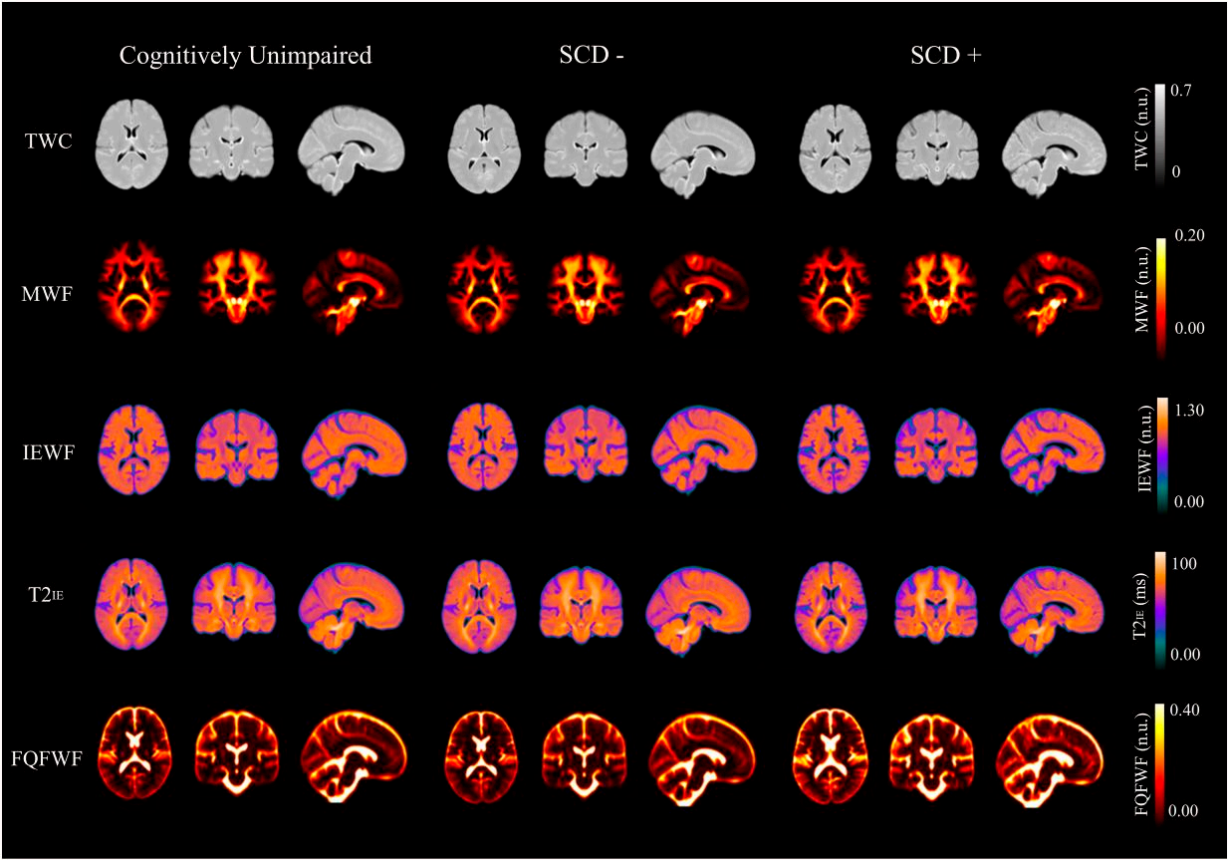
**Average maps in the MNI space of the CU (*n* = 53), SCD− (*n* = 70) and the SCD+ (*n* = 16) groups in each parameter evaluated.** TWC, total water content; MWF, myelin water fraction; IEWF, intra-extracellular water fraction; T2^IE^, T2 relaxation time of the intra-extracellular water; FQFWF, free and quasi free water fraction. The data are expressed in normalized units (n.u.) in MWF, FQFWF, TWC, IEWF and its mean T2^IE^ in milliseconds. These maps are shown only for representative purposes.

**Figure 2:**
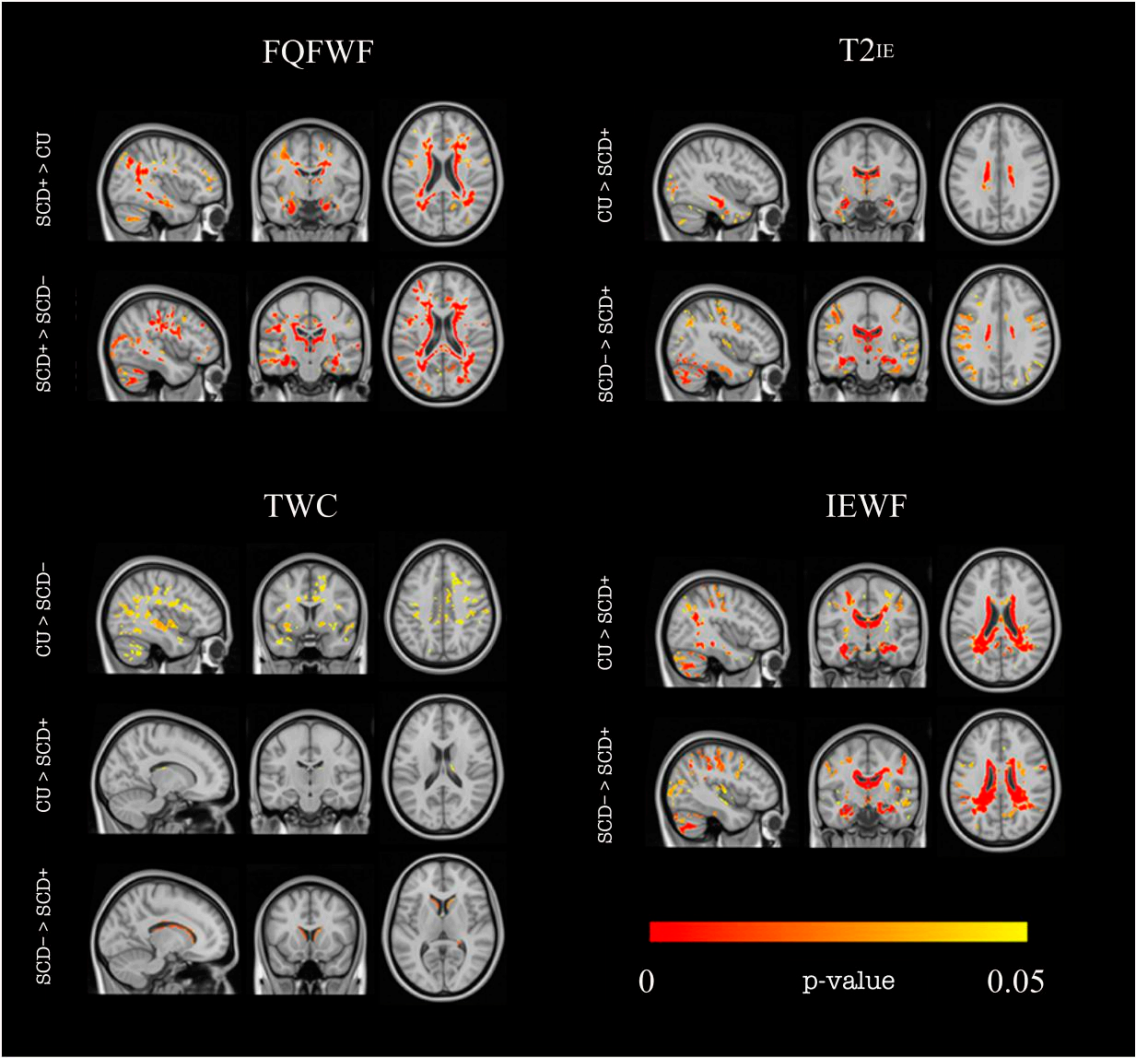
**Group differences in multi-component T2 relaxometry data revealed by ANCOVA pairwise comparisons.** Brain regions in which the voxel-wise analysis revealed significant differences between the CU (*n* = 53), SCD− (*n* = 70) and SCD+ (*n* = 16) groups for FQFWF, IEWF, T2^IE^ and TWC. FQFWF, free and quasi-free water fraction; IEWF, intra-extracellular water fraction; T2^IE^, T2 relaxation time for the intra-extracellular water; TWC, total water content.

Averaged normalized MNI maps for each parameter per group are illustrated in Fig. 1. Neuroimaging results revealed by the voxel-wise analysis are represented in Fig. 2 and group differences in grey matter regions and white matter tracts using an *F*-test are summarized in Table 3. Additional information about the clusters found to be significant in the pairwise comparisons is included in the Supplementary material.

**Table 3:** Group differences in multi-component T2 relaxometry analysis

FQFWF						
Peak of the cluster	Cluster size	MNI Coordinates (*x*, *y*, *z*)			*P*-value corrected	Involved structures
Right thalamus	115 953	18	−23	13	<0.001	Left: Caudate nucleus, hippocampus. Bilateral: Posterior corona radiata.
Right cerebellum	33 119	26	−68	−32	0.001	Bilateral: Cerebellum crus.
Left superior cerebellar peduncle	2592	−5	−38	−26	0.03	Right: Medial lemniscus, inferior cerebellar peduncle.
Left medial frontal gyrus (orbital part)	696	−11	49	−13	0.03	Left: Rectus.
Left lingual gyrus	350	−16	−78	−2	0.04	
Right inferior occipital gyrus	272	24	−101	0	0.04	
Left middle frontal gyrus	167	−31	55	18	0.04	
Right lingual gyrus	127	24	−93	−10	0.04	
Left inferior frontal gyrus (orbital part)	120	−37	38	−11	0.03	
Left middle temporal pole	117	−48	9	−30	0.04	Left: Middle temporal gyrus
Right medial frontal gyrus (orbital part)	101	13	47	−11	0.04	
Right middle cingulum	81	9	−9	50	0.04	
Right superior occipital gyrus	48	21	−90	5	0.04	
Left inferior frontal gyrus (triangular part)	32	−37	35	0	0.05	
Right superior frontal gyrus (orbital part)	10	18	55	−10	0.05	
Right middle temporal gyrus	4	65	−40	10	0.05	
**IEWF**						
Left hippocampus	92 884	−32	−15	−17	<0.001	Right: Hippocampus, thalamus. Bilateral: Caudate nucleus.
Right cerebellum crus	6821	35	−62	−27	0.005	
Right middle temporal gyrus.	332	56	−50	10	0.03	
Right lingual gyrus	285	8	−81	−1	0.04	Right: Calcarine sulcus.
Right fusiform gyrus	3	43	−45	−16	0.05	
Right middle cingulum	3	10	−44	35	0.05	
**T2^IE^**						
Left hippocampus	37 335	−32	−14	−17	0.0008	Right: Caudate nucleus, parahippocampal gyrus
Left cerebellum	2209	−4	−51	−53	0.004	Cerebellar vermis
**TWC**						
Right thalamus		13	−15	19	0.04	Right: Caudate nucleus
Left thalamus		−10	−23	18	0.04	
Left caudate nucleus		−13	4	20	0.04	
Right parahippocampal gyrus		29	−42	−3	0.05	

Grey matter regions and white matter tracts in which the *F*-test showed a significant group effect in the FQFWF, IEWF and T2^IE^ measures.

MNI, Montreal Neurological Institute. The Automated Anatomical Labelling^62^ and the Johns Hopkins University^63^ atlases were employed for anatomical reference of grey matter regions and white matter tracts, respectively.

While we did not find significant group differences in MWF, the FQFWF values (see Supplementary Table S1 in the Supplementary material) were higher in the SCD+ group as compared with CU. The peaks in the main significant clusters of this difference were seen in the cerebellum and grey matter regions including the frontal lobes (e.g. triangular part of the left inferior frontal gyrus), occipital-temporal areas (e.g. left lingual gyrus) and subcortical regions (e.g. right thalamus). These significant clusters were also seen in other grey matter regions such the medial temporal lobe (e.g. bilateral hippocampus and parahippocampal gyrus), the frontal and parietal cortex as well as other white matter tracts, including the corpus callosum, internal and external capsules, cingulum bundles and corona radiata.

The FQFWF was also higher in the SCD+ group as compared with the SCD− group (see Supplementary Table S1 in the Supplementary material). The peaks of the main significant clusters were found in temporal (left hippocampus), frontal (right medial superior frontal gyrus), occipito-temporal areas (left calcarine sulcus) and cerebellum. These clusters also involved subcortical areas and other regions of the frontal, parietal and occipital lobes as well as several white matter tracts, including the corpus callosum, corona radiata and internal/external capsules. A similar pattern of results, but in the opposite direction, was observed when evaluating the IEWF (see Supplementary Table S2 in the Supplementary material) and T2^IE^ parameters (see Supplementary Table S3 in the Supplementary material). The IEWF and T2^IE^ were higher in the CU and the SCD− groups than in the SCD+ group. Again, the overall differences were seen in the temporal areas (hippocampus, inferior, middle and superior temporal gyrus) and the frontal and parietal lobes. These clusters incorporate various subcortical regions and white matter tracts, including the corpus callosum, fornix, corona radiata and internal/external capsules.

Regarding TWC maps (see Supplementary Table S4 in the Supplementary material), the SCD+ group displayed lower TWC compared with the CU group and the SCD− group in subcortical regions (e.g. right thalamus) and medial temporal lobe areas (e.g. parahippocampal gyrus). Moreover, the SCD− displayed lower TWC compared with the CU group in several grey matter regions of the frontal (e.g. middle frontal gyrus), parietal (e.g. precuneus) and temporal lobes (e.g. parahippocampal gyrus and hippocampus).

#### ROI analysis

ROI analysis of the Alzheimer’s disease signature yielded results consistent with the voxel-wise analysis. The FQFWF was higher while the IEWF and T2^IE^ were lower in the SCD+ group as compared with the CU (FQFWF *P* = 0.025; IEWF *P* = 0.041; T2^IE^*P* = 0.046) and SCD− groups (FQFWF *P* < 0.001; IEWF *P* = 0.012; T2^IE^*P* = 0.020) groups. A follow-up ROI analysis focused on the hippocampus structure revealed significance for the FQFWF and IEWF parameters, even after adjusting for age, GDS scores and the *Z*-scores of hippocampal volume as covariates (see Table 4).

**Table 4:** Region of interest analysis of neuroimaging data

Parameter	Left hippocampus		Right hippocampus	
	SCD+ > CU	SCD+ > SCD−	SCD+ > CU	SCD+ > SCD−
FQFWF	*P* = 0.006	*P* = 0.001	*P* = 0.001	*P* = 0.001

	Left hippocampus		Right hippocampus	
	CU > SCD+	SCD− > SCD+	CU > SCD+	SCD− > SCD+
IEWF	*P* = 0.017	*P* = 0.019	*P* < 0.001	*P* = 0.001
T2^IE^	*P* = 0.784	*P* = 0.246	*P* = 1.00	*P* = 0.988

Results obtained in the follow-up ROI analysis conducted in the left and right hippocampus.

## Discussion

This study assessed differences in neurocognitive performance and microstructural tissue integrity between SCD participants with (SCD+) and without (SCD−) hippocampal atrophy relative to CU individuals. Participants underwent a comprehensive neuropsychological assessment covering several cognitive domains and also completed an MRI session that included a GRASE sequence from which five different neuroimaging metrics, namely, MWF, IEWF, T2^IE^, TWC and FQFWF, revealing different characteristics of brain integrity in grey and white matter tissues. This is the first multi-component T2-relaxometry study conducted on a cohort of individuals with SCD further categorized as SCD+ or SCD− according to the presence or absence of established features by the SCD-I working group.

There were no significant differences between the three groups in any neuropsychological tests. However, as expected, the frequency of SCCs was higher in both SCD groups as compared with control group. Additionally, we found a significant group effect in the level of depressive symptoms, although this difference did not withstand multiple comparisons correction. Increased depressive symptomatology has previously been reported in SCD.^67^ Notably, it has been demonstrated that depression and SCD are independently associated with the risk of progressing to MCI or dementia.^67,68^

Interestingly, our study did not reveal significant differences in MWF between the groups. While previous studies have reported reduced MWF in individuals with MCI,^24^ our results suggest that differences in myelin content may not be detected at preclinical stages of Alzheimer’s disease (e.g. SCD). This result requires further confirmation using complementary measures of myelin content.^69,70^ However, our investigation revealed significant differences in various MRI biomarkers of microstructural integrity. Notably, the FQFWF was higher while IEWF and T2^IE^ were lower in the SCD+ group as compared with the CU and SCD− groups. Moreover, the SCD+ and the SCD− groups showed a reduced TWC compared with the CU group and the SCD+ group showed lower TWC compared with the SCD− group. These microstructural differences were located in various cerebral structures of the frontal, parietal, temporal lobes and subcortical regions, as well as in specific white matter tracts, including the corpus callosum, the fornix, superior/anterior corona radiata and internal/external capsules. This pattern of results was also observed in brain regions critical for Alzheimer’s disease pathophysiology (i.e. the Alzheimer’s disease signature). Moreover, our secondary analysis focusing on the hippocampus structure revealed that observed differences in FQFWF and IEWF in SCD+ were not driven by volumetric differences in this medial temporal lobe region.

Elevated free water has been previously observed in individuals with MCI,^19,71-73^ and higher levels of free water have been linked to neuroinflammation in Alzheimer’s disease patients, particularly in the medial and lateral temporal lobes.^18^ This suggests that free water may serve as a valuable marker for monitoring neuroinflammation in Alzheimer’s disease, especially in these vulnerable brain regions to early neurodegeneration. Notably, neuroinflammation and glial cell activation may occur at early stages of disease progression, even before amyloid beta deposition.^74,75^ Therefore, the increased FQFWF observed in the SCD+ group suggests the presence of a higher level of neuroinflammation and neurodegeneration in these individuals, although this finding requires confirmation. The IEWF and was also lower in the SCD+ group as compared with CU and SCD−. Previous multi-component relaxometry studies have demonstrated a negative association between IEWF with increasing age.^15,16^ This age-related decline in intra axonal volume fraction (i.e. neurite density) has been consistently replicated in large-scale diffusion MRI studies using the NODDI technique, such as the UK Biobank cohort^76^ as well as in the Baltimore Longitudinal Study of Aging study.^77^ This observed lower IEWF values probably reflect accelerated degeneration of the intra- and extra-axonal compartments in the SCD+ group. However, it is important to mention that the present study evaluates relative volume fractions and reductions in the IEWF parameter can be also attributed to relative increases in MWF and FQFWF.^15,16^ Moreover, our findings indicate that the SCD+ group showed lower TWC compared with the CU and the SCD− groups, despite similar MWF. Individuals with SCD+ exhibited lower IEWF and higher FQFWF, which does not fully explain the TWC reduction.^16^ Comparing CU to SCD−, the latter showed lower TWC without significant differences in MWF, IEWF and FQFWF, suggesting global, nonspecific water content changes. Previous studies show brain water content decreases with age,^78^ but our analysis controlled for age effects. Therefore, the lower TWC found in SCD groups suggests microstructural brain tissue changes associated with SCD progression that requires further investigations. The group comparisons also revealed that the SCD+ group displayed lower T2^IE^ than the CU and SCD− groups. Lower T2^IE^ in the grey matter and white matter tracts is negatively associated with increasing age.^15^ However, as previously established, interpreting changes in T2^IE^ in terms of microstructural integrity is complicated by the involvement of multiple factors.^15^ One potential explanation for the reduced T2^IE^ in SCD+ is increased iron accumulation,^17^ which is linked to cognitive performance^79^ and has been observed in the hippocampus of Alzheimer’s disease patients.^80^

The microstructural changes observed in the white matter tracts may represent accelerated axonal neurodegeneration in the SCD+ group. The structural change could be explained by Wallerian neurodegeneration, a neuropathological process of axonal deterioration that starts with the breakdown of the cytoskeleton and ends with the fragmentation and loss of the distal axons.^81^ Thus, Wallerian neurodegeneration resulting from axonal damage could lead to white matter tract degeneration, affecting fibre integrity, myelination and axonal transport. This may lead to changes in relaxometry metrics, the formation of WMHs and disrupted neural connectivity. Several structural changes observed in the present study are predominantly located in regions where the presence of WMHs is usually observed in ageing population (e.g. the parietal lobe). WMHs are commonly attributed to ischaemia-related demyelination and axonal loss due to small vessel disease. However, they can also be linked to axonal degeneration associated with cortical Alzheimer’s disease pathology (i.e. deposition of amyloid-beta and hyperphosphorylated tau).^82,83^ The biochemical profile of WMHs in posterior parietal regions has been shown to differ between individuals with and without Alzheimer’s disease dementia. In people with Alzheimer’s disease, posterior parietal WMHs could result from degenerative axonal loss due to Wallerian degeneration, which could be triggered by cortical Alzheimer’s disease pathology, while WMHs in aged controls are related to ischaemia.^84^ Although these alternate explanations and the effects of Wallerian neurodegeneration on white matter tracts are beyond the scope of the present study, future research in this area will provide valuable insights into the complex nature of the neuropathological mechanisms underlying SCD+.

The study findings contribute to advancing our understanding of SCD and have several strengths. To our knowledge, it is the first multi-component T2-relaxometry study in SCD participants diagnosed according to the structural integrity of the hippocampus. Unlike prior investigations using 1.5T MRI scanners and relatively thick slices (8–10 mm),^85-87^ we used a 3T MRI scanner with whole-brain coverage and thinner slices (4 mm) for greater spatial accuracy. We also conducted a whole-brain voxel-wise analysis and provided supplementary evidence of microstructural differences in Alzheimer’s disease-relevant regions (i.e. Alzheimer’s disease signature). Furthermore, we assessed a broader range of neuroimaging markers (IEWF, T2^IE^, TWC and FQFWF) beyond MWF, reporting novel proxies for neuropathological processes potentially preceding widespread grey matter atrophy and white matter demyelination. This comprehensive assessment provides new insights into SCD tissue microstructure differences and complements the findings of other MRI studies. While this study raises important questions, several limitations should be addressed in subsequent research. First, the relatively small sample size as well as the limited diversity of our cohort hampers generalization of the findings. Specifically, with a sample of 53 CU individuals and 16 with SCD+, we can detect differences between CU and SCD+ participants with a magnitude of ∼0.75 SD. Likewise, with 53 CU and 70 SCD− participants, we can detect differences between CU and SCD− with a magnitude of about 0.5 SD. Additionally, the lack of a T1 map acquisition limits our ability to obtain more reliable TWC maps. Confounding factors such as WMHs load, iron deposition and magnetization transfer effects may have influenced signal interpretation. Moreover, hippocampal atrophy in the SCD+ group may indicate an elevated risk of progressing to amnestic MCI or Alzheimer’s disease.^88^ However, similar medial temporal lobe changes have been reported, albeit less prominently, in non-Alzheimer’s disease dementias like Frontotemporal Dementia^89,90^ or Dementia with Lewy Bodies.^91^ Future longitudinal studies with expanded SCD samples should incorporate blood-based Alzheimer’s disease biomarkers (e.g. amyloid β, p-tau and neurofilament light chain) to determine participants’ positions along the Alzheimer’s disease continuum, and to investigate associations between these blood-based biomarkers and MRI metrics.

In summary, in comparison to CU adults, individuals with SCD without hippocampal atrophy (SCD−) display lower TWC. Broader alterations in several grey and white matter tissue microstructure and composition were found in people with SCD and hippocampal atrophy (SCD+), including higher FQFWF as well as lower IEWF, T2^IE^ and TWC in brain areas forming part of the Alzheimer’s disease signature. This study forms the basis for further research to determine the underlying mechanisms, including neuroinflammation, intra- and extra-axonal degeneration, and increased iron deposition, that could contribute to these cerebral tissue differences in people with SCD. These investigations will have profound implications for developing early intervention to prevent cognitive impairment, while promoting brain health in individuals at risk of progressing to MCI and Alzheimer’s disease dementia.

## Supplementary Material

fcaf017_Supplementary_Data

## Data Availability

The data that support the findings of this study are available from the corresponding author, upon reasonable request. The code used in this work is publicly available at https://github.com/ejcanalesr/multicomponent-T2-toolbox.
