# Supplementary material for: Brain microstructure alterations in subjective cognitive decline: a multi-component T2 relaxometry study
Source: Brain Commun. 2025 Jan 16;7(1):fcaf017. doi: 10.1093/braincomms/fcaf017 (PMC11752640; doi:10.1093/braincomms/fcaf017)
Supplement: fcaf017_Supplementary_Data [file fcaf017_supplementary_data.pdf]

Supplementary Table 1. Pairwise comparisons in the FQFWF parameter.

FQFWF

SCD + > CU						
**Peak of the cluster**	**Cluster size**	**MNI Coordinates (x, y, z)**			**p-value corrected**	**Involved structures**
Thalamus_R	102985	18	-23	13	0.001	**Left:** Parietal_Inf, Paracentral_Lobule, Frontal_Inf_Oper, Pallidum, Frontal_Sup_Medial, Olfactory, Retrolenticular part of internal capsule, Posterior thalamic radiation include optic radiation.<br><br>**Right:** Supp_Motor_Area, Frontal_Sup_Orb, Temporal_Inf, Cingulum_Ant, Temporal_Sup, Frontal_Mid_Orb, Insula, Cuneus, Calcarine, Frontal_Inf_Tri, Precuneus, Occipital_Sup, Cingulum_cingulate_gyrus, Sagittal stratum include inferior longitudinal fasciculus and inferior fronto-occipital fasciculus.<br><br>**Bilateral:** Thalamus, Caudate, Hippocampus, Precentral, Postcentral, Angular, SupraMarginal, Frontal_Mid, Frontal_Sup, Frontal_Med_Orb, Temporal_Mid, Rectus, Rolandic_Oper, Occipital_Mid, Cingulum_Mid, Putamen, Fusiform, ParaHippocampal, Parietal_Sup, Frontal_Inf_Orb, Amygdala, Posterior corona radiata, Superior corona radiata, Anterior corona radiata, Superior longitudinal fasciculus, Uncinate fasciculus, External capsule, Cingulum hippocampus.<br><br>**Corpus Callosum:** Genu, body and splenium of corpus callosum.
Cerebellum	7294	26	-68	-32	0.01	**Right:** Cerebellum.
Lingual_L	366	-15	-79	-1	0.03	
Frontal_Inf_Tri_L	109	-34	30	16	0.04	
**SCD + > SCD -**						
**Peak of the cluster**	**Cluster size**	**MNI Coordinates (x, y, z)**			**p-value corrected**	**Involved structures**
Hippocampus_L	261160	-32	-17	-20	< 0.001	**Left:** Caudate, Paracentral_Lobule, Frontal_Inf_Tri, Heschl, Frontal_Sup_Medial, Frontal_Sup, Temporal_Pole_Sup, Parietal_Inf, Olfactory, Parietal_Sup, Posterior_corona_radiata, Superior_cerebellar peduncle, Sagittal_stratum_include inferior longitudinal fasciculus and inferior fronto-occipital fasciculus, Posterior_thalamic_radiation_include_optic_radiation.<br><br>**Right:** Thalamus, Calcarine, Cuneus, Amygdala, Cerebelum_3, Pallidum, Medial_lemniscus, Cerebral_peduncle, Cingulum_hippocampus.<br><br>**Bilateral:** Hippocampus, Fusiform, Cerebelum, Occipital_Mid, Precentral, Rolandic_Oper, Frontal_Inf_Oper, Occipital_Sup, Occipital_Inf, Lingual, Frontal_Med_Orb, Rectus, Temporal_Mid, Temporal_Inf, Cingulum_Ant, Temporal_Sup, Cingulum_Mid, Frontal_Mid, Frontal_Inf_Orb, Temporal_Pole_Mid, Putamen, Insula, Supp_Motor_Area, Postcentral, Frontal_Mid_Orb, ParaHippocampal, SupraMarginal, Precuneus, Frontal_Sup_Orb, Cingulum_Post, Angular, Inferior cerebellar_peduncle, Cingulum cingulate_gyrus, External capsule, Anterior corona radiata, Superior longitudinal fasciculus, Uncinate fasciculus, Superior corona radiata, Posterior limb of internal capsule, middle cerebellar peduncle, pontine crossing tract.<br><br>**Corpus Callosum:** Genu, body and splenium of corpus callosum,
Calcarine_L	405	-3	-70	22	0.04	**Left:** Cuneus
Frontal_Sup_Medial_R	198	14	69	4	0.04	
Cerebellum	45	17	-40	-40	0.04	

Abbreviatures can be found in Supplementary Table 5

Supplementary Table 2. Pairwise comparisons in the IEWF parameter.

IEWF

Peak of the cluster	Cluster size	MNI Coordinates (x, y, z)			p-value corrected	Involved structures
**CU > SCD+**						
Hippocampus_L	182890	-31	-14	-17	< 0.001	**Left:** Temporal_Pole_Mid, Temporal_Pole_Sup, Cerebelum_3, Occipital_Mid, Rectus, External_capsule, Superior_longitudinal_fasciculus<br><br>**Right:** Calcarine, Occipital_Sup, Paracentral_Lobule, Amygdala, Posterior_corona_radiata<br><br>**Bilateral:** Caudate, Thalamus, ParaHippocampal, Cerebelum, Lingual, Precentral, Frontal_Mid, Occipital_Inf, Postcentral, Angular, Cingulum_Mid, Precuneus, Frontal_Sup, SupraMarginal, Putamen, Fusiform, Parietal_Inf, Olfactory, Cingulum_Post, Insula, Inferior cerebellar peduncle, Medial lemniscus, Posterior limb of internal capsule.<br><br>**Corpus Callosum:** Genu, body and splenium of corpus callosum.
Temporal_Inf_R	517	47	-3	-44	0.03	**Right:** Temporal_Pole_Mid
Supp_Motor_Area_R	449	15	3	68	0.03	
Temporal_Pole_Sup_R	171	43	22	-31	0.04	
Supp_Motor_Area_L	80	-5	-15	51	0.04	
Temporal_Mid_L	21	-46	-53	14	0.05	
Parietal_Sup_L	12	-16	-73	46	0.05	
**SCD- > SCD+**						
**Peak of the cluster**	**Cluster size**	**MNI Coordinates (x, y, z)**			**p-value corrected**	**Involved structures**
Hippocampus_L	209308	-32	-15	-17	<0.001	**Left:** Frontal_Mid, Temporal_Pole_Mid, Temporal_Mid, Occipital_Mid, ParaHippocampal, Rectus, Olfactory, Cingulum_Post, Amygdala, Superior cerebellar peduncle<br><br>**Right:** Thalamus, Calcarine, Occipital_Sup, Rolandic_Oper, Insula, Frontal_Inf_Orb, Pallidum, Anterior corona radiata, Posterior limb of internal capsule, Cerebral peduncle.<br><br>**Bilateral:** Hippocampus, Caudate, Cerebellum, Postcentral, Precentral, Parietal_Inf, SupraMarginal, Lingual, Angular, Putamen, Fusiform, Superior longitudinal fasciculus, Superior corona radiata, External capsule.<br><br>**Corpus callosum:** Genu and body of corpus callosum.
Temporal_Mid_R	3130	53	-3	-20	0.02	**Right:** Temporal_Pole_Mid
Frontal_Inf_Oper_L	2915	-44	5	29	0.03	**Left:** Rolandic_Oper, Superior_longitudinal_fasciculus.
Insula_L	1622	-37	-19	13	0.02	**Left:** Temporal_Sup_L, Heschl
Occipital_Inf_R	1378	25	-89	-10	0.04	**Right:** Cuneus
Temporal_Inf_L	1153	-59	-61	-11	0.04	
Cingulum_Mid_R	591	9	-25	39	0.03	**Right:** Precuneus
Temporal_Inf_R	541	48	-3	-44	0.03	
Cingulum_Mid_L	535	-8	-40	48	0.04	**Left:** Precuneus
Cingulum_Ant_R	358	6	37	15	0.03	
Supp_Motor_Area_L	324	-5	-15	50	0.04	
Frontal_Sup_L	259	-21	15	46	0.05	
Heschl_R	187	52	-8	10	0.05	
Frontal_Inf_Oper_R	42	36	6	29	0.05	
Frontal_Inf_Tri_L	1	-34	40	12	0.05	

Abbreviatures can be found in Supplementary Table 5

Supplementary Table 3. Pairwise comparisons in the $T2^{IE}$ parameter.

$T2^{IE}$

**CU > SCD+**					
**Peak of the cluster**	**Cluster size**	**MNI Coordinates (x, y, z)**			**p-value corrected**	**Involved structures**
Hippocampus_L	51812	-31	-13	-18	<0.001	**Left:** Temporal_Inf, Temporal_Pole_Mid, Temporal_Pole_Sup, Temporal_Sup, Temporal_Mid, Precuneus, Pallidum, Amygdala, Superior cerebellar peduncle.<br><br>**Right**: Cerebelum.<br><br>**Bilateral:** ParaHippocampal, Thalamus, Fusiform, Putamen, External capsule, Medial lemniscus.<br><br>**Corpus callosum:** Genu, splenium and body of corpus callosum.
Cerebellum_L	6388	-3	-52	-53	0.003	**Left:** Superior_cerebellar_peduncle, medial lemniscus
Lingual_L	956	-8	-79	-11	0.05	
Calcarine_R	488	8	-78	9	0.04	
Temporal_Pole_Mid_R	455	55	8	-21	0.03	**Right:** Temporal_Mid_R
Temporal_Pole_Sup_R	368	45	24	-27	0.04	
Lingual_R	307	21	-95	-14	0.03	**Right:** Occipital_Inf_R
Postcentral_R	221	56	-3	21	0.03	**Right:** Precentral
Occipital_Mid_L	143	-37	-85	17	0.04	
Temporal_Inf_R	65	63	-36	-17	0.04	
Temporal_Sup_R	37	56	-5	-10	0.04	
Precuneus_R	25	15	-60	25	0.04	
Angular_R	4	34	-64	44	0.05	
Medial_lemniscus_R	2	6	-39	-31	0.05	
**SCD- > SCD+**						
**Peak of the cluster**	**Cluster size**	**MNI Coordinates (x, y, z)**			**p-value corrected**	**Involved structures**
Cerebellum	143473	-4	-51	-53	0.0005	**Left:** Pallidum, Temporal_Inf, Temporal_Pole_Mid, Occipital_Mid, Thalamus, Rectus, Temporal_Pole_Sup, Superior cerebellar peduncle, Superior longitudinal fasciculus.<br><br>**Right:** Caudate, Frontal_Inf_Orb, Occipital_Inf, Cingulum_Ant, Cuneus, Posterior thalamic_radiation_include_optic_radiation, Cingulum cingulate gyrus, Cerebral peduncle, Fornix.<br><br>**Bilateral:** Hippocampus, Cerebelum, ParaHippocampal, Putamen, Amygdala, Lingual, Calcarine, Fusiform, Olfactory, External capsule.<br><br>**Corpus callosum:** Genu_of_corpus_callosum
Postcentral_L	22144	-41	-20	44	0.01	**Left:** Precentral, Angular, SupraMarginal, Parietal_Inf, Frontal_Mid, Insula, Temporal_Sup, Heschl, Rolandic_Oper, Frontal_Inf_Oper, Temporal_Mid, Frontal_Sup.
Postcentral_R	8050	56	-3	21	0.007	**Right:** Precentral, Frontal_Inf_Oper, Insula, Rolandic_Oper, Heschl.
Temporal_Pole_Mid_R	7674	55	9	-22	0.01	**Right:** Temporal_Mid, Temporal_Sup, Temporal_Pole_Sup
Angular_R	2901	42	-61	34	0.03	**Right:** Occipital_Mid
Temporal_Inf_R	766	60	-60	-2	0.02	
Parietal_Inf_R	520	30	-40	54	0.02	**Right:** Parietal_Sup.
Occipital_Sup_R	336	21	-88	38	0.04	
SupraMarginal_R	119	40	-33	46	0.05	
Precuneus_R	115	5	-47	40	0.04	**Right:** Cingulum_Mid
Frontal_Inf_Tri_L	93	-39	42	12	0.03	
Parietal_Sup_L	65	-31	-69	54	0.04	
Supp_Motor_Area_R	63	15	4	67	0.04	

Abbreviatures can be found in Supplementary Table 5

Supplementary Table 4. Pairwise comparisons in the TWC parameter.

TWC

**CU > SCD-**						
**Peak of the cluster**	**Cluster size**	**MNI Coordinates (x, y, z)**			**p-value corrected**	**Involved structures**
Left middle occipital gyrus	38204	-41	-70	18	0.03	**Left:** Temporal_Sup, Hippocampus, Thalamus, Lingual, Cerebelum, Fusiform, Temporal_Mid, Precuneus, Occipital_Mid, Angular, Cerebelum_8, Occipital_Sup, Insula, ParaHippocampal, Occipital_Inf, Amygdala, Calcarine, Putamen, SupraMarginal, Temporal_Pole_Sup, Cuneus, Pallidum.<br><br>**Bilateral:** Vermis
Left middle frontal gyrus	37334	-27	47	19	0.04	Left: Cingulum_Mid, Postcentral, Frontal_Mid, Frontal_Sup_Medial, Caudate, Paracentral_Lobule, Rolandic_Oper, Parietal_Inf, Parietal_Sup, Precentral, Cingulum_Ant.<br><br>Bilateral: Frontal_Inf_Tri, Frontal_Inf_Oper.
Right middle temporal gyrus	24746	50	-2	-19	0.03	**Left:** Lingual, Precuneus, Temporal_Sup, Temporal_Mid, Fusiform, Thalamus, Cerebelum, Temporal_Inf, Occipital_Inf, Pallidum, Hippocampus, ParaHippocampal, Temporal_Pole_Sup, Caudate<br><br>**Bilateral:** Cerebellum
Right paracentral lobule	6430	9	-31	66	0.04	**Right:** Postcentral, SupraMarginal, Paracentral_Lobule, Parietal_Sup, Supp_Motor_Area, Cingulum_Mid.
Right precentral gyrus	3655	30	-2	52	0.04	**Right:** Middle frontal gyrus.
Right inferior frontal gyrus (orbital)	3045	46	51	-11	0.04	**Right:** Middle frontal gyrus (orbital)
Right insula	709	41	14	-4	0.04	
Right superior frontal gyrus (orbital)	292	26	56	-4	0.04	
Left inferior temporal gyrus	205	-57	-55	-6	0.05	
Right rolandic operculum	173	60	-5	15	0.05	
Right superior occipital gyrus	132	22	-74	33	0.05	**Right:** Middle occipital gyrus
Left supplementary motor area	116	-7	17	58	0.05	
Right calcarine sulcus	77	14	-91	7	0.05	
Right rectus	26	7	50	-16	0.05	
Left superior frontal gyrus	18	-22	65	8	0.05	
**SCD- > SCD+**						
**Peak of the cluster**	**Cluster size**	**MNI Coordinates (x, y, z)**			**p-value corrected**	**Involved structures**
Right thalamus	7226	13	-15	19	0.009	**Left:** Caudate nucleus.
**CU > SCD+**						
**Peak of the cluster**	**Cluster size**	**MNI Coordinates (x, y, z)**			**p-value corrected**	**Involved structures**
Right thalamus	66	16	-23	17	0.04	
Right parahippocampal gyrus	6	29	-42	-3	0.05	

Abbreviatures can be found in Supplementary Table 5

Supplementary Table 5. Abbreviatures of grey matter regions included in the Automated Anatomical Labelling atlas.

**Grey matter anatomical abbreviatures**	
Precentral	Precentral gyrus
Frontal_Sup	Superior frontal gyrus
Frontal_Sup_Orb	Superior frontal gyrus (orbital part)
Frontal_Mid	Middle frontal gyrus
Frontal_Mid_Orb	Middle frontal gyrus (orbital part)
Frontal_Inf_Oper	Inferior frontal gyrus (opercular part)
Frontal_Inf_Tri	Inferior frontal gyrus (triangular part)
Frontal_Inf_Orb	Inferior frontal gyrus (orbital part)
Rolandic_Oper	Rolandic operculum
Supp_Motor_Area	Supplementary motor area
Olfactory	Olfactory bulb
Frontal_Sup_Medial	Superior medial frontal gyrus
Frontal_Med_Orb	Medial frontal gyrus (orbital part)
Rectus	Rectus
Insula	Insula
Cingulum_Ant	Anterior cingulate gyrus
Cingulum_Mid	Midcingulate gyrus
Cingulum_Post	Posterior cingulate gyrus
Hippocampus	Hippocampus
ParaHippocampal	Parahippocampal gyrus
Amygdala	Amygdala
Calcarine	Calcarine sulcus
Cuneus	Cuneus
Lingual	Lingual gyrus
Occipital_Sup	Superior occipital gyrus
Occipital_Mid	Middle occipital gyrus
Occipital_Inf	Inferior occipital gyrus
Olfactory	Olfactory cortex
Fusiform	Fusiform gyrus
Postcentral	Postcentral gyrus
Parietal_Sup	Superior parietal lobe
Parietal_Inf	Inferior parietal lobe
SupraMarginal	Supramarginal gyrus
Angular	Angular gyrus
Precuneus	Precuneus
Paracentral_Lobule	Parancetral lobule
Caudate	Caudate
Putamen	Putamen
Pallidum	Pallidum
Thalamus	Thalamus
Heschl	Heschl
Temporal_Sup	Superior temporal gyrus
Temporal_Pole_Sup	Temporal lobe of the superior temporal gyrus
Temporal_Mid	Middle temporal gyrus
Temporal_Pole_Mid	Temporal pole of the middle temporal gyrus
Temporal_Inf	Inferior temporal gyrus
Cerebellum	Cerebellum
